# ^18^F-FDG PET/CT radiomics signature and clinical parameters predict progression-free survival in breast cancer patients: A preliminary study

**DOI:** 10.3389/fonc.2023.1149791

**Published:** 2023-03-10

**Authors:** Xiaojun Xu, Xun Sun, Ling Ma, Huangqi Zhang, Wenbin Ji, Xiaotian Xia, Xiaoli Lan

**Affiliations:** ^1^ Department of Nuclear Medicine, Union Hospital, Tongji Medical College, Huazhong University of Science and Technology, Wuhan, China; ^2^ Hubei Province Key Laboratory of Molecular Imaging, Wuhan, China; ^3^ Key Laboratory of Biological Targeted Therapy of the Ministry of Education, Wuhan, China; ^4^ He Kang Corporate Management (SH) Co. Ltd, Shanghai, China; ^5^ Department of Radiology, Affiliated Taizhou Hospital of Wenzhou Medical University, Taizhou, Zhejiang, China

**Keywords:** ^18^F-fluorodeoxyglucose, positron emission tomography/computed tomography, breast cancer, radiomics signature, progression-free survival

## Abstract

**Introduction:**

This study aimed to investigate the feasibility of predicting progression-free survival (PFS) in breast cancer patients using pretreatment ^18^F-fluorodeoxyglucose positron emission tomography/computed tomography (FDG PET/CT) radiomics signature and clinical parameters.

**Methods:**

Breast cancer patients who underwent ^18^F-FDG PET/CT imaging before treatment from January 2012 to December 2020 were eligible for study inclusion. Eighty-seven patients were randomly divided into training (n = 61) and internal test sets (n = 26) and an additional 25 patients were used as the external validation set. Clinical parameters, including age, tumor size, molecular subtype, clinical TNM stage, and laboratory findings were collected. Radiomics features were extracted from preoperative PET/CT images. Least absolute shrinkage and selection operators were applied to shrink feature size and build a predictive radiomics signature. Univariate and multivariate Cox proportional hazards models and Kaplan-Meier analysis were used to assess the association of rad-score and clinical parameter with PFS. Nomograms were constructed to visualize survival prediction. C-index and calibration curve were used to evaluate nomogram performance.

**Results:**

Eleven radiomics features were selected to generate rad-score. The clinical model comprised three parameters: clinical M stage, CA125, and pathological N stage. Rad-score and clinical-model were significantly associated with PFS in the training set (*P*< 0.01) but not the test set. The integrated clinical-radiomics (ICR) model was significantly associated with PFS in both the training and test sets (*P*< 0.01). The ICR model nomogram had a significantly higher C-index than the clinical model and rad-score in the training and test sets. The C-index of the ICR model in the external validation set was 0.754 (95% confidence interval, 0.726–0.812). PFS significantly differed between the low- and high-risk groups stratified by the nomogram (*P* = 0.009). The calibration curve indicated the ICR model provided the greatest clinical benefit.

**Conclusion:**

The ICR model, which combined clinical parameters and preoperative ^18^F-FDG PET/CT imaging, was able to independently predict PFS in breast cancer patients and was superior to the clinical model alone and rad-score alone.

## Introduction

Breast cancer is the most prevalent cancer and leading cause of cancer death in women (1). Although adjuvant therapy had improved survival, 5-year overall relative survival rates for locally advanced and metastatic breast cancer were 55% and 18%, respectively (2). Determining predictors of survival is essential for developing individualized treatment strategies and improving prognosis.

High intratumoral heterogeneity in breast cancer is associated with worse prognosis (3, 4) and is difficult to ascertain using typical invasive biopsy techniques. Clinicopathological parameters including age, tumor size and stage, and metastasis status are conventional prognostic factors for breast cancer (5). However, clinical outcomes may vary because of highly heterogeneity and these factors alone may not provide accurate prognostic information.

Imaging has considerable potential in guiding breast cancer treatment. ^18^F-fluorodeoxyglucose positron emission tomography/computed tomography (^18^F-FDG PET/CT) is widely used for initial staging, monitoring recurrence and treatment response, and assessing prognosis (6–12). However, conventional PET using semi-quantitative parameters does not fully reflect internal tumoral characteristics, which limits the forecasting accuracy (13–15).

Radiomics can noninvasively characterize intratumoral heterogeneity by extracting multiple high-dimensional quantitative features from medical images. This approach has the ability to reveal the biological behavior of the entire tumor and has great potential to predict prognosis (16–19).

In breast cancer, ^18^F-FDG PET/CT radiomics has been used to classify molecular subtype, predict treatment response, and assess prognosis (18, 20–28). Several previous studies have investigated prediction of breast cancer prognosis using PET/CT radiomics features (24, 26–29). However, these studies were small or lacked external validation datasets. In addition, prediction models that combine imaging and clinical factors are more accurate than those that use imaging and clinical factors alone (30).

Therefore, this study aimed to develop and validate model nomograms to predict progression-free survival (PFS) in breast cancer patients using clinical parameters and PET/CT radiomics features.

## Methods

### Study population

This retrospective study was approved by Ethics Committee of the Union Hospital of Tongji Medical College of Huazhong University of Science and Technology, and the requirement for written informed consent was waived. We retrospectively analyzed 87 female breast cancer patients (51.8 ± 12.9 years, range 25.0-81.0) who underwent ^18^F-FDG PET/CT imaging before treatment in our institution (first center) from January 2012 to December 2020. Patients were randomly divided into a training set (n = 61) and internal test set (n = 26). A total of additional 25 patients (female, 55.9 ± 11.1 years, range 35.0-82.0) from the first center (Wuhan Union Hospital) and second center (Taizhou Hospital) were collected as an external validation set.

**Figure 1:**
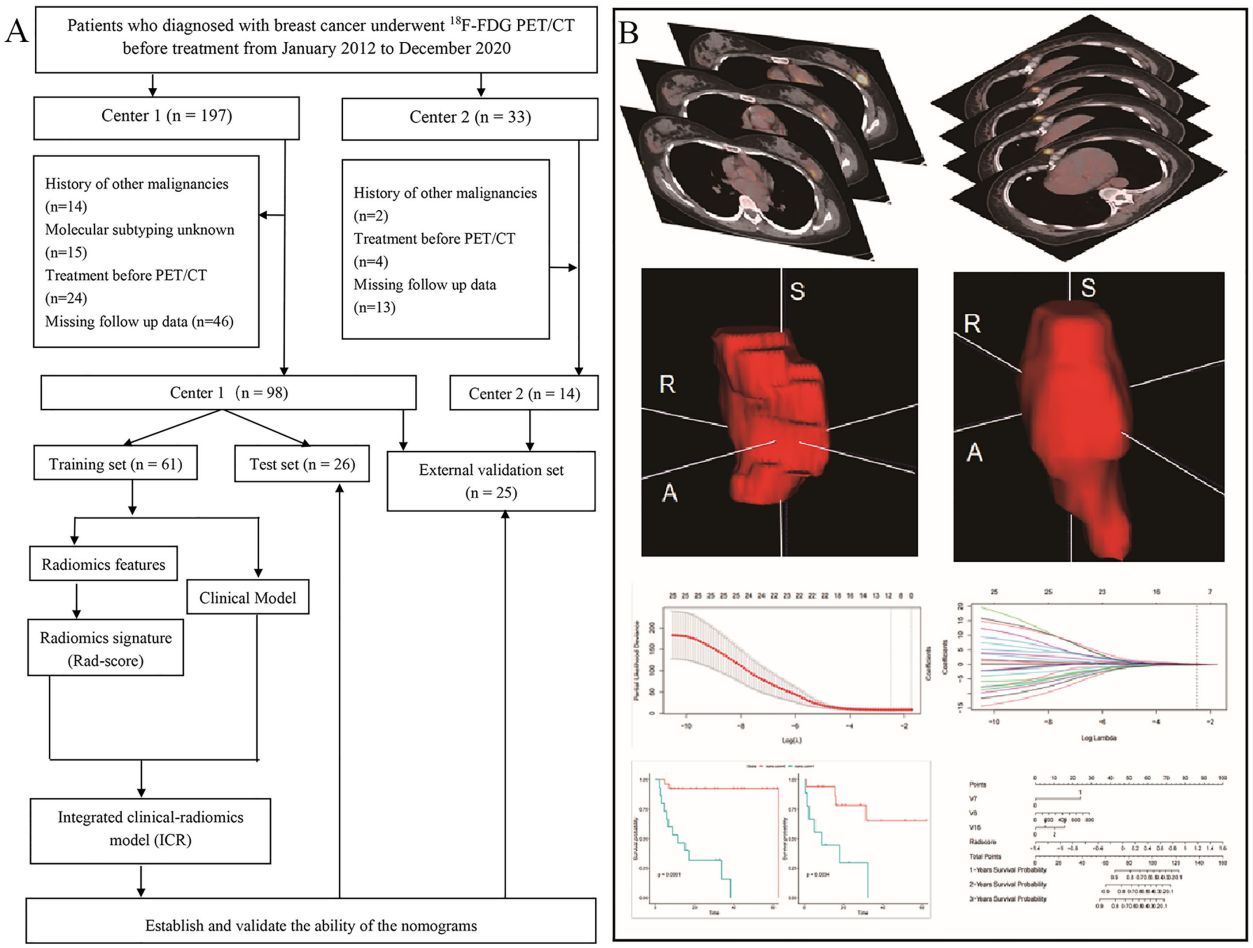
Patient screening and positron emission tomography/computed tomography radiomics analysis. **(A)** Study flowchart. **(B)** Schematic representation of the radiomics analysis workflow.

Patients who underwent treatment before PET/CT and those with a history of other cancer, unknown molecular subtype, or blood glucose concentration > 11.1 mmol/L before ^18^F-FDG injection were excluded. We also excluded patients with missing data and those lost to follow-up. A study flowchart is shown in **Figure 1A**.

### Clinical evaluation

Clinical parameters, including age, tumor size, molecular subtype, TNM stage, and concentrations of pretreatment carcinoembryonic antigen (CEA), carbohydrate antigen 125 (CA125), and carbohydrate antigen 15-3 (CA15-3) were recorded.

### ^18^F-FDG PET/CT imaging

^18^F-FDG was synthesized using ^18^F produced by a cyclotron (MINItrace^®^, GE Healthcare, Milwaukee, WI, USA) with radiochemical purity >95%. All patients were required to fast for at least 6 hours before ^18^F-FDG injection. Blood glucose concentration was measured prior to injection (only patients with concentration ≤ 11.1 mmol/L were included). Intravenous ^18^F-FDG (3.70-5.55 MBq/kg) was administered and PET/CT was performed approximately 60 minutes later using a Discovery VCT^®^ system (GE Healthcare). PET/CT acquisition and reconstruction parameters are shown in the Additional file 1.

### Delineation and segmentation of PET/CT images

The radiomics workflow is shown in **Figure 1B**. ^18^F-FDG PET/CT digital imaging and communications in medicine images were retrieved and loaded into ITK-SNAP software (www.itksnap.org) for manual segmentation. Before PET image segmentation, 40% maximum standardized uptake value threshold mapping was calculated using LIFEx (https://www.lifexsoft.org/). Delineation of the region of interest (ROI) was performed manually by a nuclear medicine physician with 3 years of experience (XX). All ROIs were segmented by two nuclear medicine physicians with more than 15 years of experience (XS and XL). Repeatability of parameters extracted from the ROIs segmented by these two physicians was evaluated using the interclass correlation coefficient (ICC), and reserved the parameters with ICC coefficient greater than 0.6.

### Radiomics features extraction

The PyRadiomics feature package imported into Anaconda prompt software (github.com/Radiomics/pyradiomics, version 4.2.0) was used to extract radiomics features according to the feature guide of the image biomarker standardization initiative. The categories and number of extracted radiomics features are detailed in the Additional file 1.

### Features screening and models construction

Continuous variables were concentrated and standardized. Eighty-seven patients were randomly divided into training and test sets at a ratio of 7:3.

#### Radiomics signature (Rad-score)

The minimal redundancy maximal relevance (mRMR) algorithm (31), which can improve the accuracy of feature selection and classification, was used to select the initial features in the training set. The least absolute shrinkage and selection operator (LASSO) was used to screen features. Parameters corresponding to the minimum penalty and weight coefficients were selected to construct the radiomics signature. The radiomics signature was calculated for each patient by a linear combination of selected features weighted by their respective coefficients.

#### Clinical model

In the training set, univariate and multivariate Cox proportional hazard regression were used to analyze and screen clinical features. Features were selected using the minimum Akaike information criterion to avoid overfitting. Furthermore, associations between the clinical parameters and PFS in the training set were evaluated and then verified in the test set.

#### Integrated clinical-radiomics model

Clinical features and rad-score were used to create a multivariate Cox proportional hazard regression model.

### Evaluation of model performance

To evaluate model performance, the radiomics nomogram, clinical nomogram, and ICR model nomogram were built in the training set, then evaluated in the internal test set, and verified in the external validation set.

The concordance index (C-index), which measures the proportion of the predicted results consistent with the actual results in all patient pairs, was used to evaluate discriminating ability. C-index between 0.50 and 0.70 indicated poor accuracy, while a value between 0.71 and 0.90 indicated moderate accuracy; values above 0.90 indicated high accuracy (32). Bootstrap verification (2000 Bootstrap resampling) was performed on the training and test sets to calculate the relative corrected C-index.

A calibration curve was used to validate the ICR model nomogram performance, which used bootstrap resampling to evaluate the original data. Integrated area under curve (iAUC) of the receiver operating characteristic (ROC) curve was used to evaluate predictive performance of the combined model.

### Outcome evaluation

Follow-up was conducted by clinic visits or telephone. The study endpoint was PFS. PFS was defined as time from the date of initial PET/CT to the date of disease progression, recurrence, death from any cause, or last follow-up. Patients who did not have progression/recurrence at the date of their last clinical follow-up were considered as a censored data.

### Statistical analysis

Categorical variables were compared using Pearson’s chi-square test. Continuous variables were compared using the unpaired two-tailed Students t-test assuming or the Wilcoxon rank sum test as appropriate. *P*< 0.05 was considered significant. ROC curve analysis was used to determine the rad-score threshold and divide patients into high- and low-risk groups. Survival was analyzed using the Kaplan–Meier method. Survival curves were compared using the log-rank test. Statistical analyses were performed using R software version 3.6.4 (www.rproject.org). The packages used included lattice, use this, devtools, tidyverse, caret, publish, survival, glmnet, ggpubr, survminer, rolr, survIDINRI, survAUC, rms, dca.)

## Results

### Patient characteristics

A total of 112 newly diagnosed breast cancer patients were included for analysis. The clinicopathological characteristics of the training (n = 61) and internal test (n = 26) sets patients are shown in **Table 1**. Characteristics of the 25 patients in external validation set are summarized in **Supplementary Table S1**.

**Table 1:** Clinicopathological characteristics of patients in the training and test sets.

Characteristics	Overall cohort (n = 87)	Training set (n = 61)	Test set (n = 26)	*P* value*
		No. (70%)	No. (30%)	
Age (y) Median(range)	50.0 (25.0-81.0)	50.0 (25.0-78.0)	47.5 (28.0-81.0)	0.921
≤50y	46 (52.9)	31 (50.8)	15 (57.7)	0.724
>50y	41 (47.1)	30 (49.2)	11 (42.3)	
Tumor size				0.096
≤2cm	43 (49.4)	35 (57.4)	8 (30.8)	
>2cm	44 (50.6)	26 (42.6)	18 (69.2)	
SUVmax				0.820
Median(range)	6.9 (1.3-25.2)	7.0 (1.6-25.2)	6.1 (1.3-17.5)	
Subtype				0.151
Luminal A	21 (24.1)	17 (27.9)	4 (15.4)	
Luminal B	33 (37.9)	19 (31.1)	14 (53.8)	
HER-2	19 (21.9)	13 (21.3)	6 (23.1)	
Triple negative	14 (16.1)	12 (19.7)	2 (7.7)	
cT**^†^**				0.413
T1	38 (43.7)	30 (49.2)	8 (30.8)	
T2	39 (44.8)	24 (39.3)	15 (57.7)	
T3	4 (4.6)	3 (4.9)	1 (3.8)	
T4	6 (6.9)	4 (6.6)	2 (7.7)	
cN**^†^**				0.543
N0	31 (35.6)	20 (32.8)	11 (42.3)	
N1	14 (16.1)	12 (19.7)	2 (7.7)	
N2	17 (19.6)	12 (19.7)	5 (19.2)	
N3	25 (28.7)	17 (27.8)	8 (30.8)	
cM**^†^**				0.159
M0	58 (66.7)	44 (72.1)	14 (53.8)	
M1	29 (33.3)	17 (27.9)	12 (46.2)	
pT**^‡^**				0.448
T1	32 (36.8)	25 (41.0)	7 (26.9)	
T2	42 (48.3)	26 (42.6)	16 (61.5)	
T3	5 (5.7)	4 (6.6)	1 (3.9)	
T4	8 (9.2)	6 (9.8)	2 (7.7)	
pN**^‡^**				0.800
N0	29 (33.3)	19 (31.1)	10 (38.5)	
N1	15 (17.3)	12 (19.7)	3 (11.5)	
N2	16 (18.4)	11 (18.1)	5 (19.2)	
N3	27 (31.0)	19 (31.1)	8 (30.8)	
pM**^‡^**				0.218
M0	60 (69.0)	45 (73.8)	15(57.7)	
M1	27 (31.0)	16 (26.2)	11(42.3)	
CA125				0.890
Positive	26 (29.9)	19 (31.1)	7 (26.9)	
Negative	61 (70.1)	42 (68.9)	19 (73.1)	
CA15-3				0.082
Positive	27 (31.0)	15 (24.6)	12 (46.2)	
Negative	60 (69.0)	46 (75.4)	14 (53.8)	
CEA				0.301
Positive	19 (21,8)	11 (18.0)	8 (30.8)	
Negative	68 (78.2)	50 (82.0)	18 (69.2)	

^*^ The difference of clinicopathological characteristics between the training set and test set.

**^†^** c-stage indicates clinical stage as determined by positron emission tomography/computed tomography.

**^‡^** p-stage indicates stage as determined by pathology

CA125, Carbohydrate antigen 125; CA15-3, Carbohydrate antigen 15-3; CEA, Carcinoembryonic antigen; SUVmax, Maximum standardized uptake value

### PFS

All patients underwent breast-conserving surgery or mastectomy. The details of adjuvant therapy (including radiotherapy, chemotherapy and endocrine therapy) are shown in **Table 1**. During follow up, thirty of the 87 patients in the training and internal test sets (34.5%) experienced recurrence or progression. Among these, mean PFS was 25.4 ± 19.4 months (range, 0.3-64.4) and median PFS was 20.4 months. Seven of the 25 external validation set patients (28.0%) experienced recurrence or progression. Among these, mean PFS was 17.3 ± 3.3 months (range, 2.1-70.1) and median PFS was 11.7 months.

### Radiomics signature construction and testing

**Figure 2:**
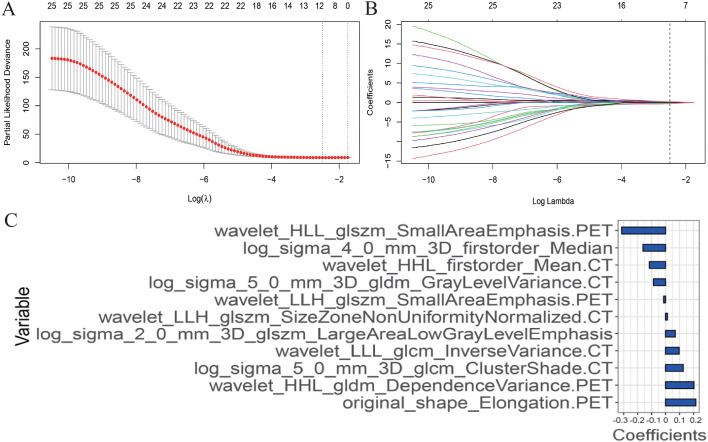
Radiomics features selection using the least absolute shrinkage and selection operator (LASSO) Cox regression model. **(A)** The partial likelihood deviance (PLD) curve was plotted versus log (λ), where λ is the tuning parameter. Solid vertical lines represent PLD ± standard error (SE). The dotted vertical lines are drawn at the optimal values by using the minimum criteria and 1−SE criteria. Tuning parameter (λ) selection in the LASSO model used 10-fold cross-validation *via* minimum criteria. A value λ = 1.210 with log (λ) = 0.083 was chosen. **(B)** LASSO coefficient profiles of the positron emission tomography and computed tomography radiomics features. A coefficient profile plot was produced against the log (λ) sequence. The optimal tuning parameter resulted in 11 non-zero coefficients. **(C)** The weight ratio coefficients of the 11 features included in the radiomics model.

Based on the training set, a total of 1920 PET/CT radiomics features were extracted. A LASSO Cox regression was performed to achieve regression coefficient compression and select variables (**Figures 2A, B**).

After screening, 11 radiomics features were included in the final model: original_shape_Elongation.PET, wavelet_HHL_gldm_Dependence Variance.PET, log_sigma_5_0_mm_3D_glcm_ClusterShade.CT, wavelet_LLL_glcm_Inverse Variance.CT, log_sigma_2_0_mm_3D_glszm_Large Area Low GrayLevelEmphasis, wavelet_LLH_glszm_SizeZoneNonUniformityNormalized.CT, wavelet_LLH_glszm_SmallAreaEmphasis.PET, log_sigma_5_0_mm_3D_gldm_GrayLevelVariance.CT, wavelet_HHL_firstorder_Mean.CT, log_sigma_4_0_mm_3D_firstorder_Median and wavelet_HLL_glszm_SmallAreaEmphasis.PET. Rad-score was calculated for each patient using a linear combination of selected features weighted by their respective coefficients as follows (**Figure 2C**):


$$\begin{array}{l} \text{Rad-score}=\;0.216308907988105\;\times \\ \text{original}\_\text{shape}\_\text{Elongation}.\text{ PET }+0.20247294159178\text{ x}\\ \text{wavelet}\_\text{HHL}\_\text{gldm}\_\text{DependenceVariance}.\text{ PET }+\;0.126675894088301\;\times \\ \text{log}\_\text{sigma}\_5\_0\_\text{mm}\_3\text{D}\_\text{glcm}\_\text{ClusterShade}.\text{ CT }+0.0971773019410209\;\times \\ \text{wavelet}\_\text{LLL}\_\text{glcm}\_\text{Inverse Variance}.\text{ CT }+0.0687564202753143\text{ x}\\ \text{log}\_\text{sigma}\_2\_0\_\text{mm}\_3\text{D}\_\text{glszm}\_\text{LargeAreaLowGrayLevelEmphasis }+\;0.013847068926288\\ \text{wavelet}\_\text{LLH}\_\text{glszm}\_\text{SizeZoneNonUniformityNormalized}.\text{ CT }-\;0.0133830861893992\text{ x}\\ \text{wavelet}\_\text{LLH}\_\text{glszm}\_\text{SmallAreaEmphasis}.\text{ PET }-\;0.0870828100344496\;\times \\ \text{log}\_\text{sigma}\_5\_0\_\text{mm}\_3\text{D}\_\text{gldm}\_\text{GrayLevelVariance}.\text{ CT }-\;0.115345107379321\;\times \\ \text{wavelet HHL}\_\text{firstorder}\_\text{Mean}.\text{ CT }-\;0.161665805895483\text{ x}\\ \text{log}\_\text{sigma}\_4\_0\_\text{mm}\_3\text{D}\_\text{firstorder}\_\text{Median }–\;0.314539923888585\;\times \\ \text{walvet}\_\text{HLL}\_\text{glszm}\_\text{SmallAreaEmphasis}.\text{ PET} \end{array}$$

**Figure 3:**
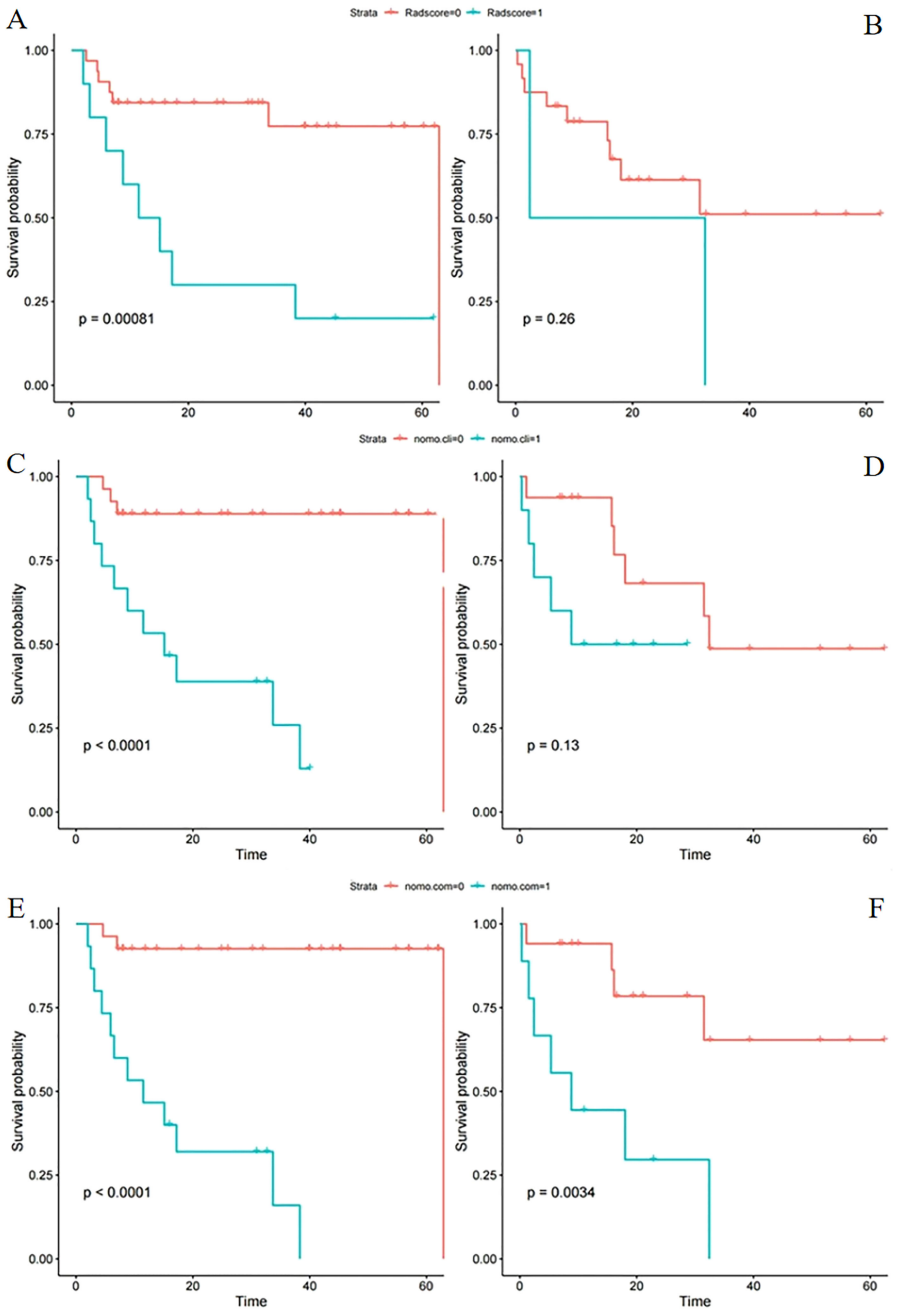
Kaplan–Meier survival analysis of different models in the training and test sets. Progression-free survival according to the radiomics score **(A, B)**, clinical model **(C, D)**, and integrated clinical radiomics model **(E, F)** for patients in the training set (L) and test set (R). **(A, B)** A significant association of the radiomic signature with PFS was shown in the training set but not the test set. **(C, D)** A significant association of the clinical model with PFS was shown in the training set but not the test set. **(E, F)** A significant association of the integrated model with PFS was shown in both the training and test sets (cli indicates clinical; com indicates combined clinical and rad-score).

The scores of patients in the training and test sets were calculated through the constructed radiomics signature. Patients were divided into high- and low-risk groups based on the optimal cutoff determined by ROC curve analysis. In the training set, PFS was significantly shorter in patients with a higher rad-score (*P<* 0.001; **Figure 3A**). In the test set, the difference was not significant (*P* = 0.260, **Figure 3B**).

### Clinical features model construction and testing

The results of univariate Cox regression analyses in the training and test sets are shown in **Supplementary Table S2**. Three parameters were included (clinical M stage, CA125, pathological N stage) in the multivariate Cox model. In the training set, multivariate analysis showed that clinical M stage (hazard ratio [HR] 7.67; 95% confidence interval [CI], 1.98-29.77; *P* = 0.003) and CA125 (HR 1.00; 95% CI, 1.00-1.01; *P* = 0.011) were independent predictors of PFS; pathological N stage was not (HR 1.46; 95% CI, 0.89-2.40; *P* = 0.138). However, in the test set, clinical M stage (HR 3.12; 95% CI, 0.76-12.77; *P* = 0.113), CA125 (HR 1.00; 95% CI, 1.00-1.00; *P* = 0.377), and pathological N stage (HR 1.53; 95% CI, 0.91-2.56; *P* = 0.108) were not independent predictors of PFS (**Table 2**).

**Table 2:** Multivariate cox regression analysis in the training and test sets.

Characteristics	Training set		Test set	
	HR (95% CI)	*P* value	HR (95% CI)	*P* value
Clinical M stage	7.67 (1.98-29.77)	**0.003**	3.12 (0.76-2.77)	0.113
CA125	1.00 (1.00-1.01)	**0.011**	1.00 (1.00-1.00)	0.377
Pathological N stage	1.46 (0.89-2.40)	0.138	1.53 (0.91-2.56)	0.108

CA125, Carbohydrate antigen 125; CI, Confidence interval; HR: Hazard ratio. *P*<0.05 was considered statistically significant and presented as bold values

The constructed clinical model was used to calculate the clinical score for each patient. PFS significantly differed between the high- and low risk groups in the training set (*P*< 0.001; **Figure 3C**) but not the test set (*P* = 0.130; **Figure 3D**).

### ICR model construction and testing

An ICR model including rad-score and clinical parameters was established based on stepwise multivariate Cox analysis. In the training set, rad-score (HR 6.52; 95% CI, 1.56-27.36; *P* = 0.010) was an independent predictor of PFS, but clinical M stage (HR 3.84; 95% CI, 0.83-17.63; *P* = 0.084), CA125 (HR 1.002; 95% CI, 1.002-1.004; *P* = 0.124) and pathological N stage (HR 1.34; 95% CI, 0.79-2.28; *P* = 0.280) were not. None of the variables were independent predictors in the test set (**Table 3**).

**Table 3:** Multivariate cox regression of the integrated clinical-radiomics model in the training and test sets.

Characteristics	Training set		Test set	
	HR (95% CI)	*P* value	HR (95% CI)	*P* value
Clinical M stage	3.84 (0.83-17.63)	0.084	4.02 (0.91-17.70)	0.066
CA125	1.002 (1.002-1.004)	0.124	1.011 (0.99-1.04)	0.599
Pathological N stage	1.34 (0.79-2.28)	0.280	1.90 (1.04-3.45)	0.036
Rad-score	6.52 (1.56-27.36)	**0.010**	6.67 (0.77-57.81)	0.085

CA125, Carbohydrate antigen 125; CI, Confidence interval; HR: Hazard ratio. *P*<0.05 was considered statistically significant and presented as bold values.

The ICR model equation was as follows:


$$h\left(t,x\right)=h_{0}\left(t\right){\text{e}}^{\left(1.345\times \text{Initial M staging}+0.0019\times \text{CA}125+0.293\times \text{pathological N staging}+1.87\times \text{Rad}-\text{score}\right)}$$

**Figure 4:**
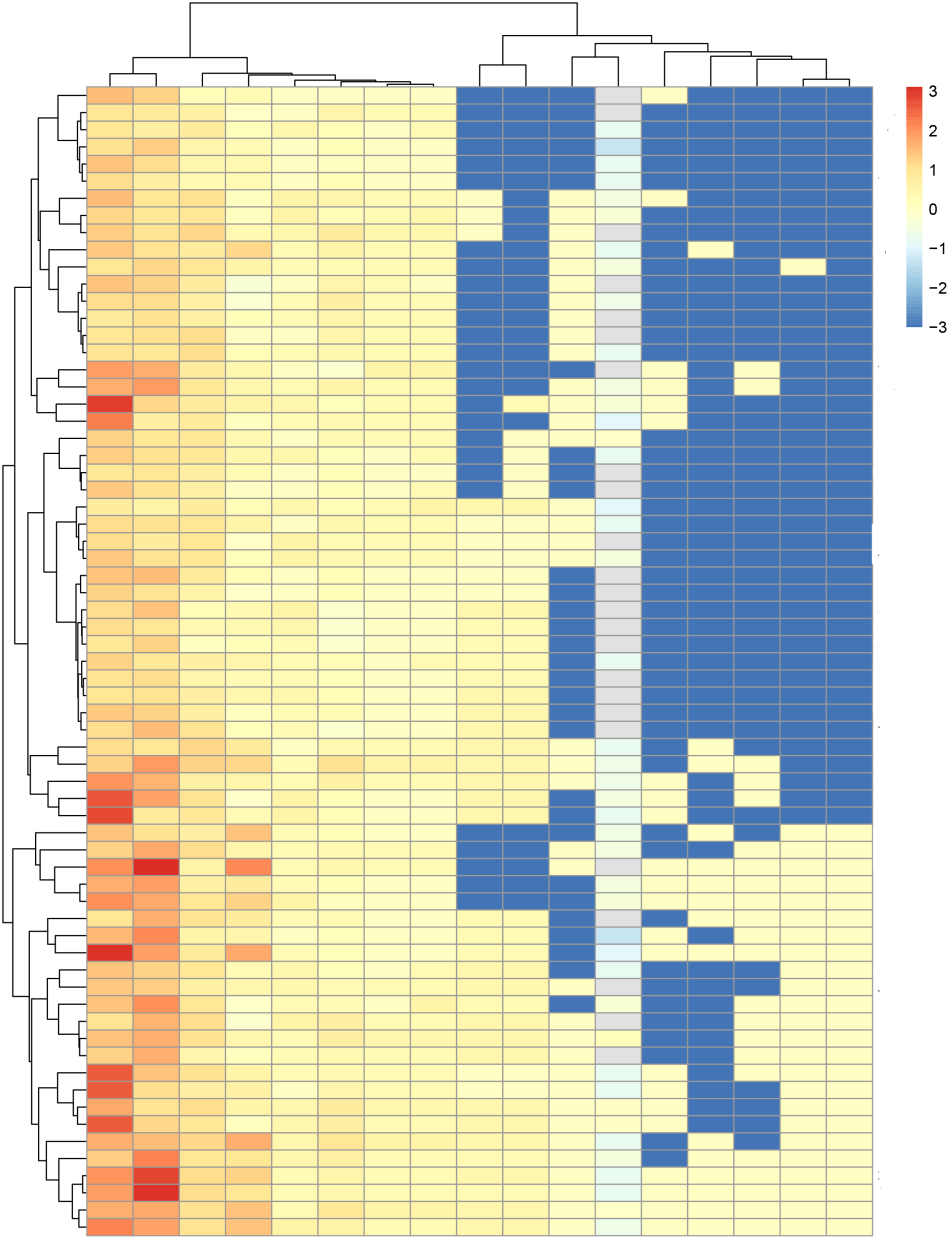
Hierarchically clustered heatmap of the feature correlation matrix. Features with an inter-correlation above the selected threshold (≥0.7) were removed from the dataset.

S significantly differed between the high- and low risk groups in both the training and test sets (*P*< 0.001 and *P* = 0.003, respectively; **Figures 3E, F**). The ICR model was examined for correlation between parameters using Spearman analysis; parameters with the same trend were examined through unsupervised cluster analysis. Hierarchically clustered heatmap of the feature correlation matrix is shown in **Figure 4**. Features with an inter-correlation above the selected threshold (≥0.7) were removed from the dataset.

### Model performance evaluation

**Figure 5:**
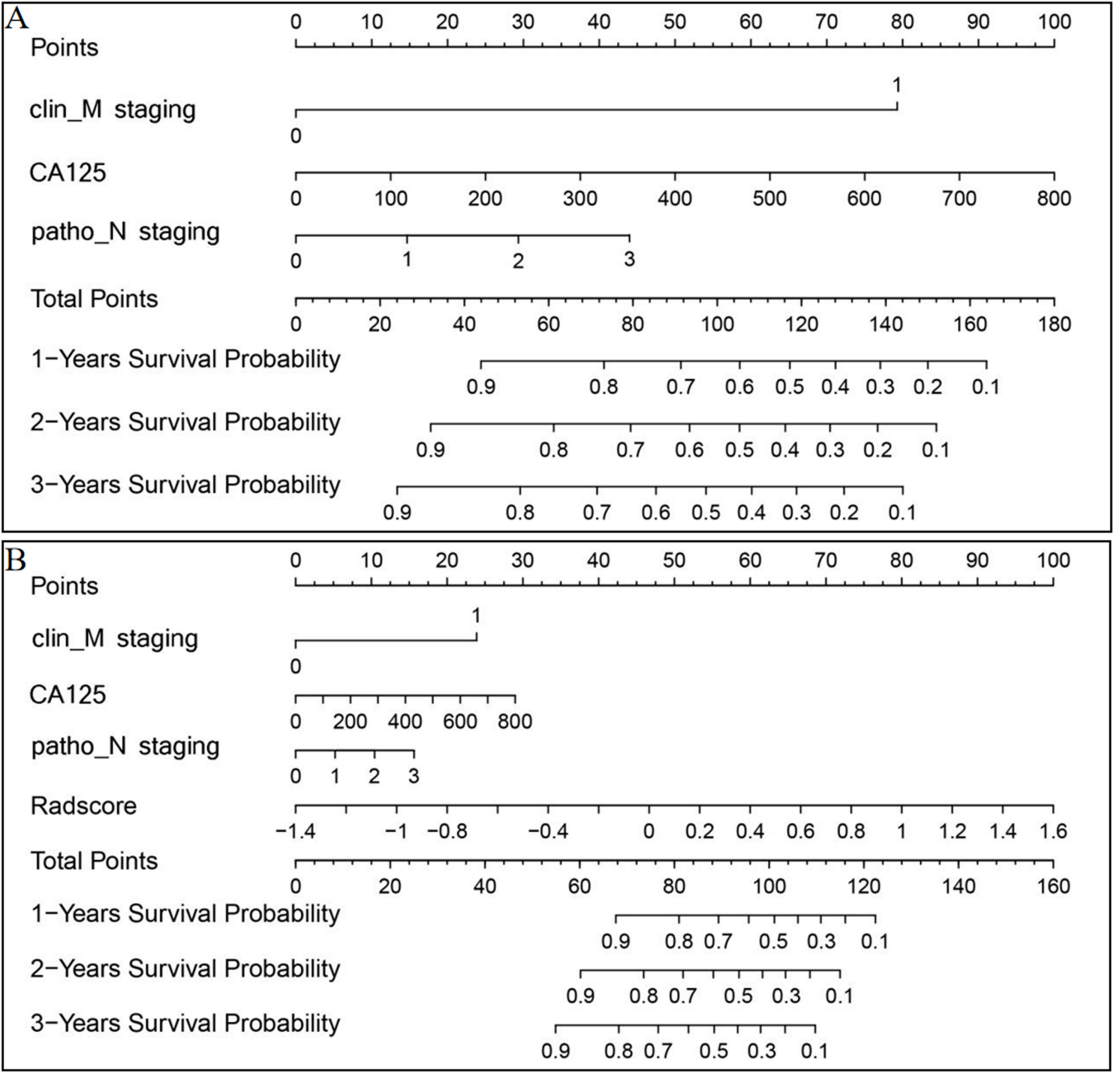
Clinical **(A)** and ICR **(B)** model nomograms to predict survival using the training set. Drawing a vertical line to the points’ axis from specific variables determined the number of points toward the probability of progression-free survival. The process was repeated for each variable and the points for each risk factor were added. The final total was then located on the total points axis.

Nomograms for rad-score and the clinical and ICR models were developed (**Figures 5A, B**) and predictive performance was evaluated. In the training and test sets, the C-indices for rad-score were 0.777 (95% CI, 0.712-0.833) and 0.626 (95% CI, 0.597-0.755), respectively. Corresponding C-indices for the clinical nomogram were 0.790 (95% CI, 0.754-0.872) and 0.714 (95% CI, 0.632-0.774), respectively, and those for the ICR nomogram were 0.845 (95% CI, 0.793-0.912) and 0.758 (95% CI, 0.723-0.801), respectively (**Supplementary Table S3**). In both the training and test sets, the ICR model achieved the best prediction accuracy.

**Figure 6:**
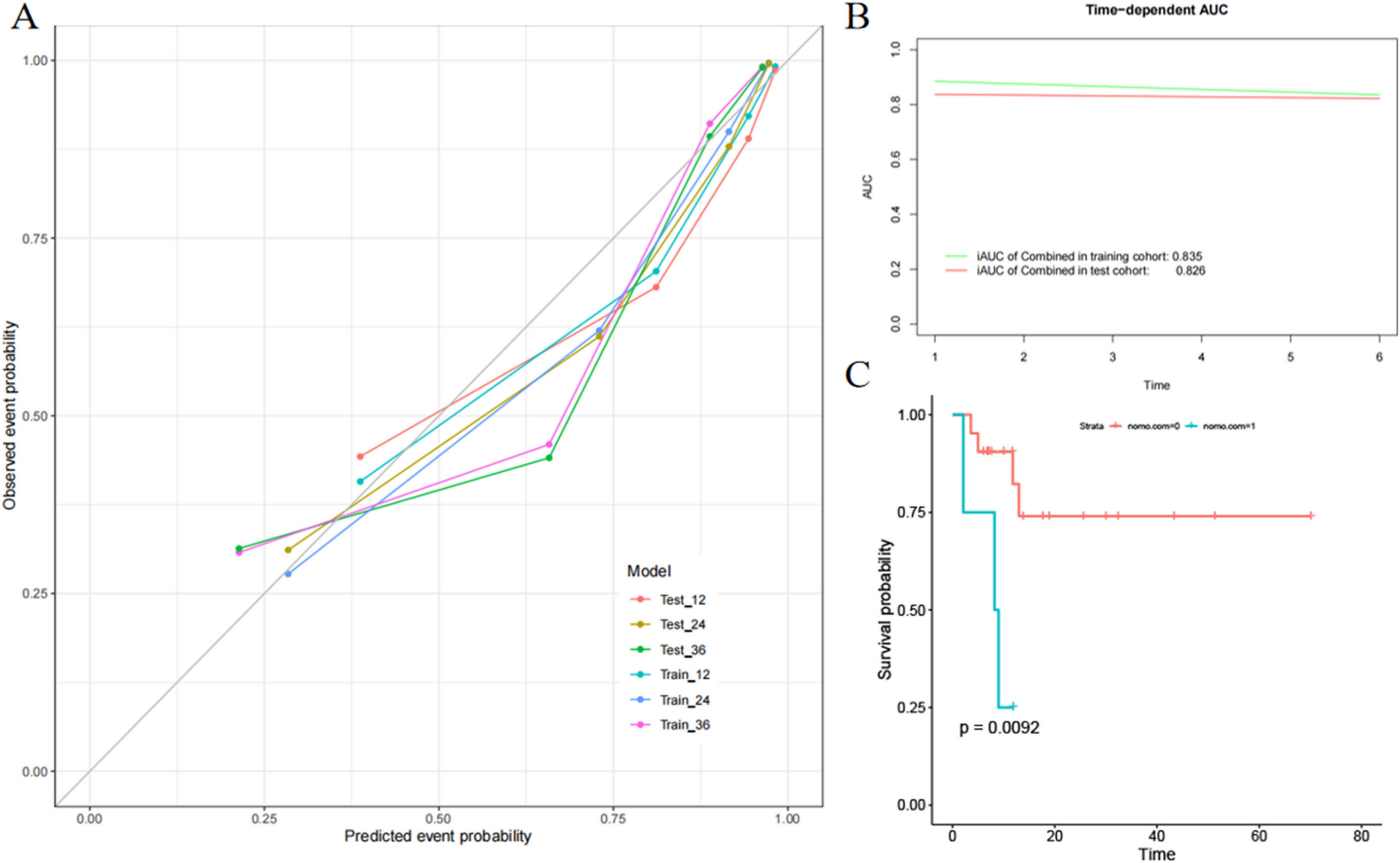
Evaluation of the integrated clinical radiomics (ICR) model performance. **(A)** Calibration curves of the ICR nomogram for progression-free survival. **(B)** The integrated area under the curve (weighted mean of the area under the curve over the follow-up period) was used to measure model performance in survival prediction. **(C)** Kaplan–Meier survival analysis according to the ICR model for patients in the external validation set.

In the ICR model, mean iAUC in the training and test sets was 0.835 and 0.826, respectively (**Figure 6A**). To assess consistency between predicted and actual PFS, calibration curves of the ICR model in the training and test sets were plotted (**Figure 6B**). Agreement between the predicted and observed curves was good and the bias curves in both sets were near to the ideal line.

### Models constructed based on PET or CT alone

Rad-score was also constructed based on PET and CT images alone. The regression coefficient and variable selection are shown in the **Supplementary Figure S1**. Compared with PET/CT, the performance of the rad-score and ICR model as constructed by PET and CT alone was worse (**Supplementary Figures S2**, **S3**).

### Performance in the external validation set

To fully evaluate the ICR model performance, external validation was performed. The ICR model nomogram yielded a favorable C-index value in the external validation set (0.754; 95% CI, 0.726-0.812). PFS significantly differed between the low- and high-risk groups stratified by the nomogram (**Figure 6C**), suggesting good prognostic value (*P* = 0.009).

## Discussion

In this study, we retrospectively analyzed newly diagnosed breast cancer patients and developed models based on ^18^F-FDG PET/CT imaging and clinical parameters before treatment to predict PFS. Through internal and external validation, we demonstrated that the ICR model could predict PFS well. Moreover, the ICR model was significantly better than models comprised solely of clinicopathologic variables or PET/CT imaging data. This emphasizes and supports the importance of multidisciplinary collaboration and indicates that integration of clinical parameters and PET/CT imaging features can better predict breast cancer progression and improve prognosis. Our model provides a simple and easily used tool for breast cancer patients with strong heterogeneity, aiding clinicians in rapidly evaluating the probability of progression. However, it still needs to be validated in large prospective studies.

The ICR model was able to predict PFS of breast cancer patients with a higher C-index and better calibration than the radiomics signature or clinical model. It took advantage of the synergy of rad-score and clinical features, which concurred well with the results of previous studies (3, 22, 24, 26, 30, 33–35). Our results also showed that the addition of rad-score to clinical data might be used for risk assessment.

PET/CT radiomics has shown considerable potential for prognostication in breast cancer patients. In our study, rad-score comprised four PET radiomics features and seven CT features. Most were derived texture features, including GLCM, GLDM, and GLSZM. These features reflect the interaction between adjacent pixels, which are appropriate for quantifying textural heterogeneity of tumors. The prognostic value of these features in breast cancer has been reported and emphasized in previous studies (22, 30, 36, 37).

In this study, rad-score was an independent predictor of PFS in the training set but not the test set, although rad-score was higher in patients who experienced tumor progression. The results in previous studies that examined PET/CT radiomics in breast cancer prognostication were also inconsistent. However, most yielded promising findings, suggesting that rad-score is an independent prognostic factor (3, 22, 27). Similar to our study, Groheux et al. (38) found that entropy value derived from PET/CT imaging could predict event-free survival of locally advanced breast cancer (*P*< 0.050); however, in multivariate analysis, PET texture analysis had no added value. The likely reason was that, first, to avoid and reduce the over-fitting effect, the radiomics features were de-redundant and removing impurity when constructing the rad-score model in the training set. Second, due to our small sample size, the amount of data in the model training process was small, the performance might be reduced. Furthermore, our model might be affected by heterogeneity between different datasets and research methodologies.

Similar to rad-score, the clinical model alone did not independently predict PFS in the test set, which suggests that clinical parameters alone do not accurately reflect heterogeneity and the risk of progression. Among clinical parameters, N and M stage are well-known conventional prognostic factors (11, 39). In addition, ^18^F-FDG PET/CT has the ability to detect distant metastases, which adds to its value in prognostic evaluation.

This study had several limitations. It was retrospective in design and had both a small sample size and relatively short follow-up. In addition, ROI delineation and calculation of imaging parameters were not automatically performed. Prospective large-scale multicenter studies are warranted to validate our models and expand the application of PET/CT radiomics in breast cancer.

In conclusion, our ICR model, which combines clinical parameters with radiomics score, shows considerable promise in predicting PFS in breast cancer patients and deserves further study.

## Data availability statement

The raw data supporting the conclusions of this article will be made available by the authors, without undue reservation.

## Ethics statement

The studies involving human participants were reviewed and approved by the Committee of Union Hospital, Tongji Medical College. The patients/participants provided their written informed consent to participate in this study.

## Author contributions

Conceptualization: XL. Data curation: XXu and HZ. Formal analysis: XXu and LM. Investigation: XXu and HZ. Methodology: XXu and LM. Software: XXu and LM. Resources: WJ and XL. Visualization: XXu and LM. Project administration: XS, XXia, and XL. Writing-original draft preparation: XXu. Writing-review and editing: XXu, LM, XXia, and XL. All authors contributed to the article and approved the submitted version.
