# Supplementary material for: 18F-FDG PET/CT radiomics signature and clinical parameters predict progression-free survival in breast cancer patients: A preliminary study
Source: Front Oncol. 2023 Mar 10;13:1149791. doi: 10.3389/fonc.2023.1149791 (PMC10036789; doi:10.3389/fonc.2023.1149791)
Supplement: Supplementary file 1 [file DataSheet_1.docx]

**Supplementary material**

**Supplementary Data.**

**PET/CT acquisition and reconstruction parameters**

Both CT and PET were acquired from the top of the skull to the upper thighs. CT scout image was used to locate the scanning position prior to scanning. The CT attenuation correction acquisition parameters were as follows: voltage, 120 kV; tube current, 110 mAs; and slice thickness, 3.75 mm. Then, PET data were acquired using 2 min/bed position. All PET images were corrected for attenuation using the acquired CT data. The original images were reconstructed using the iterative method after attenuation correction. Then, PET, CT, and PET/CT fusion images were obtained in the transverse, sagittal, and coronal planes (Xeleris workstation; GE Healthcare). All PET and CT images were resampled as 1*1*1 mm^3^ in MATLAB 2016a (Math Works, Natick, MA, USA) to provide same spatial information (thickness, slice, and interlamellar space).

**Extracted radiomics features**

The extracted radiomics features included first order, shape, gray level co-occurrence matrix (GLCM), gray level run length matrix (GLRLM), gray level size zone matrix (GLSZM), and gray level dependence matrix(GLDM). The original image was processed using wavelet and Laplacian of Gaussian (LoG) filters. Then, radiomics features were extracted from the original PET/CT image and the filtered image. The shape feature was extracted from the original image; other types of features were extracted from the original image and the derived image at the same time. Finally, 960 PET and 960 CT radiomics features were obtained.

**Table S1** Clinicopathological characteristics of patients in the external validation set

| Characteristics | Overall cohort  (n = 25/%) | Center 1 (n = 11) | | Center 2 (n =14) | | | *P*value |
| --- | --- | --- | --- | --- | --- | --- | --- |
|  |  | Progress (n = 3) | Non-progress (n = 8) | | Progress (n = 4) | Non-progress (n = 10) |  |
| Age (y) | 55.9±11.1 | 55.0±10.1 | 55.0±13.8 | | 56.3±17.3 | 56.7±9.0 | 0.965 |
| Tumor size |  |  |  | |  |  |  |
| ≤ 2cm | 16 (64.0%) | 1 | 6 | | 4 | 5 | 0.636 |
| > 2cm | 9 (36.0%) | 2 | 2 | | 0 | 5 |  |
| SUVmax | 6.6±3.8 | 7.5±2.5 | 5.4±2.0 | | 4.1±2.5 | 8.3±4.9 | 0.412 |
| Subtype |  |  |  | |  |  | 0.944 |
| Luminal A | 7 (28.0%) | 1 | 3 | | 1 | 2 |  |
| Luminal B | 7 (28.0%) | 1 | 2 | | 1 | 3 |  |
| HER-2 | 5 (20.0%) | 0 | 2 | | 0 | 3 |  |
| Triple negative | 6 (24.0%) | 1 | 1 | | 2 | 2 |  |
| cT**^*^** |  |  |  | |  |  | 0.916 |
| T1 | 16 (64.0%) | 1 | 6 | | 4 | 5 |  |
| T2 | 6 (24.0%) | 0 | 2 | | 0 | 4 |  |
| T3 | 1 (4.0%) | 1 | 0 | | 0 | 0 |  |
| T4 | 2 (8.0%) | 1 | 0 | | 0 | 1 |  |
| cN**^*^** |  |  |  | |  |  | 0.149 |
| N0 | 10 (40.0%) | 0 | 3 | | 1 | 6 |  |
| N1 | 4 (16.0%) | 1 | 2 | | 0 | 1 |  |
| N2 | 3 (12.0%) | 1 | 1 | | 0 | 1 |  |
| N3 | 8 (32.0%) | 1 | 2 | | 3 | 2 |  |
| cM**^*^** |  |  |  | |  |  | 0.548 |
| M0 | 18 (72.0%) | 1 | 8 | | 1 | 8 |  |
| M1 | 7 (28.0%) | 2 | 0 | | 3 | 2 |  |
| pN^†^ |  |  |  | |  |  | 0.355 |
| N0 | 9 (36.0%) | 0 | 2 | | 1 | 6 |  |
| N1 | 5 (20.0%) | 1 | 3 | | 0 | 1 |  |
| N2 | 2 (8.0%) | 0 | 1 | | 0 | 1 |  |
| N3 | 9 (36.0%) | 2 | 2 | | 3 | 2 |  |
| pM^†^ |  |  |  | |  |  | 0.017 |
| M0 | 17 (68.0%) | 1 | 8 | | 1 | 7 |  |
| M1 | 8 (32.0%) | 2 | 0 | | 3 | 3 |  |
| CA125 |  |  |  | |  |  | 0.032 |
| Positive | 14 (56.0%) | 2 | 1 | | 2 | 9 |  |
| Negative | 11 (44.0%) | 1 | 7 | | 2 | 1 |  |

Pathological T stage CEA, and CA153 in the external validation set were affected by a confidentiality agreements and further statistical analyses were not performed. **^*^** c-stage indicates clinical stage as determined by positron emission tomography/computed tomography, ^†^ p-stage indicates stage as determined by pathology.

Center 1: Union Hospital, Tongji Medical College, Huazhong University of Science and Technology; Center 2: Taizhou Hospital, Zhejiang Province

CA 125: Carbohydrate antigen 125; SUVmax: Maximum standardized uptake value

**Table S2** Univariate cox regression analysis of the training and test sets

| Characteristics | Training set (n = 61) | | *P* value | Test set (n = 26) | | *P* value |
| --- | --- | --- | --- | --- | --- | --- |
|  | Progress (n =19) | Non-progress (n = 42) |  | Progress (n = 11) | Non-progress (n = 15) |  |
| Age, year | 53.6±13.8 | 51.2±12.8 | 0.633 | 54.4±14.6 | 49.6±11.2 | 0.107 |
| Tumor size | 3.2±2.1 | 2.1±1.1 | **0.024** | 3.9±2.9 | 2.9±2.1 | **0.032** |
| SUVmax | 10.5±5.2 | 6.3±3.7 | **0.020** | 9.6±4.7 | 6.4±4.0 | **0.044** |
| Median (range) | 9.2 (4.0-25.2) | 5.5 (1.6-18.4) |  | 8.7 (4.1-17.5) | 5.3 (1.3-14.4) |  |
| Subtype |  |  | 0.053 |  |  | 0.256 |
| Luminal A | 5 (26.3) | 12 (28.6) |  | 0 (0.0) | 4 (26.7) |  |
| Luminal B | 4 (21.1) | 15 (35.7) |  | 6 (54.5) | 8 (53.3) |  |
| HER-2 | 6 (31.5) | 7 (16.7) |  | 4 (36.4) | 2 (13.3) |  |
| Triple negative | 4 (21.1) | 8 (19.0) |  | 1 (9.1) | 1 (6.7) |  |
| cT**^*^** |  |  | 0.100 |  |  | 0.155 |
| T1 | 7 (36.8) | 23 (54.7) |  | 2 (18.2) | 6 (40.0) |  |
| T2 | 7 (36.8) | 17 (40.5) |  | 7 (63.6) | 8 (53.3) |  |
| T3 | 3 (15.8) | 0 (0.0) |  | 1 (9.1) | 0 (0.0) |  |
| T4 | 2 (10.6) | 2 (4.8) |  | 1 (9.1) | 1 (6.7) |  |
| cN**^*^** |  |  | **0.018** |  |  | 0.154 |
| N0 | 3 (15.8) | 17 (40.5) |  | 2 (18.2) | 9 (60.0) |  |
| N1 | 3 (15.8) | 9 (21.4) |  | 1 (9.1) | 1 (6.7) |  |
| N2 | 3 (15.8) | 9 (21.4) |  | 3 (27.3) | 2 (13.3) |  |
| N3 | 10 (52.6) | 7 (16.7) |  | 5 (45.4) | 3 (20.0) |  |
| cM**^*^** |  |  | **0.0002** |  |  | 0.053 |
| M0 | 8 (42.1) | 36 (85.7) |  | 4 (36.4) | 10 (66.7) |  |
| M1 | 11 (57.9) | 6 (14.3) |  | 7 (63.6) | 5 (33.3) |  |
| pT^†^ |  |  | 0.053 |  |  | 0.299 |
| T1 | 5 (26.3) | 20 (47.6) |  | 2 (18.2) | 5 (33.3) |  |
| T2 | 8 (42,1) | 18 (42.9) |  | 7 (63.6) | 9 (60.0) |  |
| T3 | 3 (15.8) | 1 (2.4) |  | 1 (9.1) | 0 (0.0) |  |
| T4 | 3 (15.8) | 3 (7.1) |  | 1 (9.1) | 1 (6.7) |  |
| pN^†^ |  |  | **0.029** |  |  | 0.346 |
| N0 | 2 (10.5) | 17 (40.5) |  | 3 (27.3) | 7 (46.7) |  |
| N1 | 3 (15.8) | 9 (21.4) |  | 0 (0.0) | 3 (20.0) |  |
| N2 | 4 (21.1) | 7 (16.7) |  | 3 (27.3) | 2 (13.3) |  |
| N3 | 10 (52.6) | 9 (21.4) |  | 5 (45.4) | 3 (20.0) |  |
| pM^†^ |  |  | **0.0002** |  |  | 0.053 |
| M0 | 8 (42.1) | 37 (88.1) |  | 5 (45.5) | 10 (66.7) |  |
| M1 | 11 (57.9) | 5 (11.9) |  | 6 (54.5) | 5 (33.3) |  |
| CA125 |  |  | **0.012** |  |  | 0.415 |
| Positive | 10 (52.6) | 9 (21.4) |  | 4 (36.4) | 3 (20.0) |  |
| Negative | 9 (47.4) | 33 (78.6) |  | 7 (63.6) | 12 (80.0) |  |
| CA15-3 |  |  | **0.013** |  |  | 0.710 |
| Positive | 7 (36.8) | 8 (19.0) |  | 7 (63.6) | 5 (33.3) |  |
| Negative | 12 (63.2) | 34 (81.0) |  | 4 (36.4) | 10 (66.7) |  |
| CEA |  |  | **0.003** |  |  | 0.973 |
| Positive | 5 (26.3) | 6 (14.3) |  | 5 (45.5) | 3 (20.0) |  |
| Negative | 14 (73.7) | 36 (85.7) |  | 6 (54.5) | 12 (80.0) |  |
| Adjuvant chemotherapy |  |  | **0.041** |  |  | 0.158 |
| Yes | 18 (94.7) | 30 (71.4) |  | 8 (72.7) | 14 (93.3) |  |
| No | 1 (5.3) | 12 (28.6) |  | 3 (27.3) | 1 (6.7) |  |
| Adjuvant radiotherapy |  |  | 0.100 |  |  | 0.951 |
| Yes | 12 (63.2) | 17 (40.5) |  | 6 (54.5) | 8 (53.3) |  |
| No | 7 (36.8) | 25 (59.5) |  | 5 (45.5) | 7 (46.7) |  |
| Adjuvant endocrinotherapy |  |  | 0.849 |  |  | 0.781 |
| Yes | 9 (47.4) | 21 (50.0) |  | 5 (45.5) | 6 (40.0) |  |
| No | 10 (52.6) | 21 (50.0) |  | 6 (54.5) | 9 (60.0) |  |

^*^ c-stage indicates clinical stage as determined by positron emission tomography/computed tomography. **^†^** p-stage indicates stage as determined by pathology.

CA125: Carbohydrate antigen 125; CA15-3: Carbohydrate antigen 15-3; CEA: Carcinoembryonic antigen; SUVmax: Maximum standardized uptake value

**Table S3** Comparison of prognostic performance among the clinical model, radiomics signature and integrated clinical-radiomic model

| Sets | Radiomics signature | | | | Clinical model | | | | Integrated clinical-radiomics model | | | |
| --- | --- | --- | --- | --- | --- | --- | --- | --- | --- | --- | --- | --- |
|  | C-index | Likelihood | Wald | Log-rank | C-index | Likelihood | Wald | Log-rank | C-index | Likelihood | Wald | Log-rank |
| Training set | 0.777 | 0.000 | 0.000 | 0.000 | 0.790 | 0.000 | 0.000 | 0.000 | 0.845 | 0.000 | 0.000 | 0.000 |
| Test set | 0.626 | 0.080 | 0.200 | 0.260 | 0.714 | 0.020 | 0.020 | 0.130 | 0.758 | 0.003 | 0.005 | 0.003 |


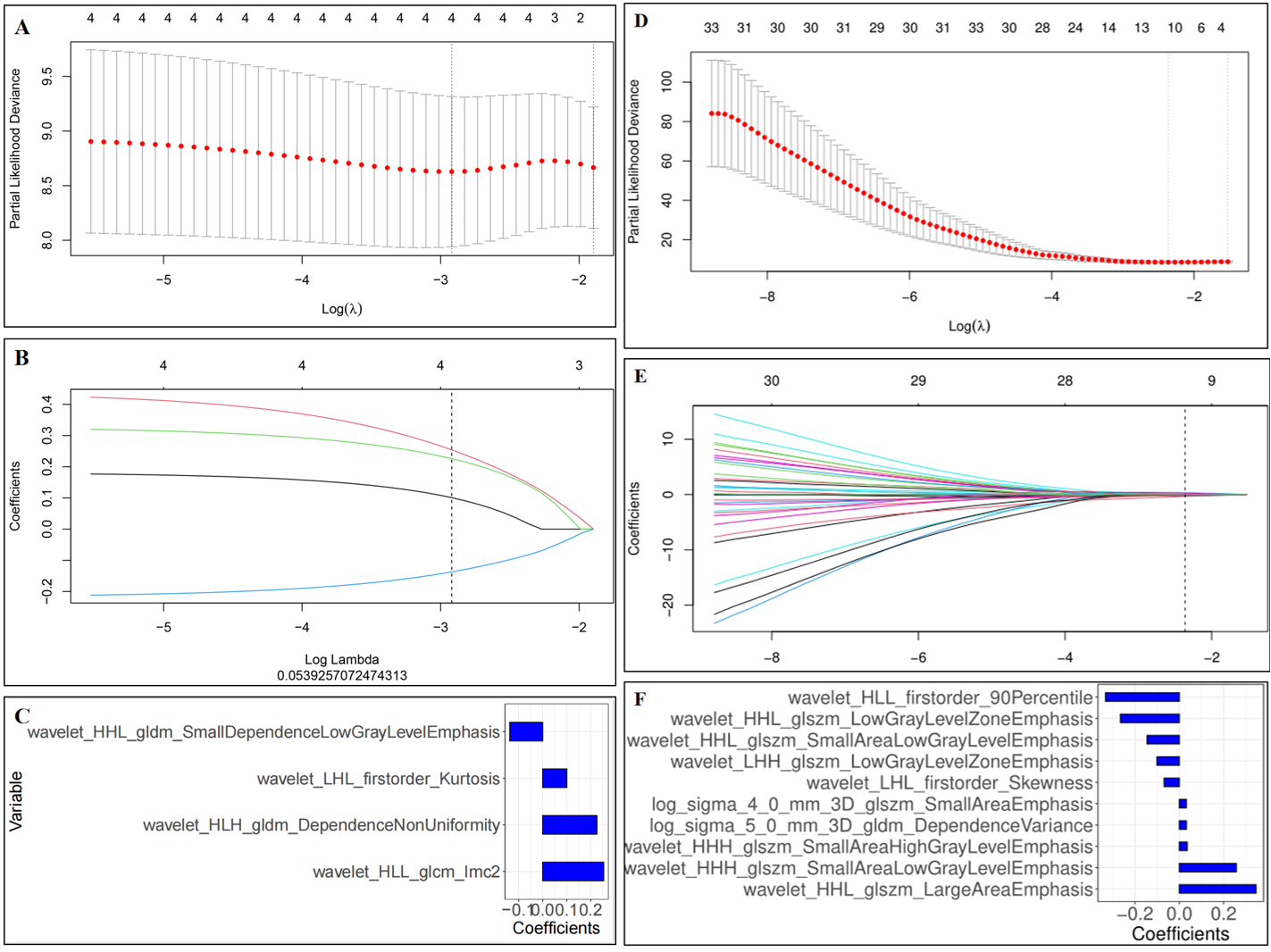


**Figure S1.** Radiomics features selection using least absolute shrinkage and selection operator (LASSO) Cox regression (positron emission tomography, A-C; computed tomography, D-F). **(A, D)** The partial likelihood deviance curve was plotted and determined the tuning parameter (λ). **(B, E)** LASSO coefficient profiles of the radiomics features. **(C, F)** Weight ratio coefficients of radiomic features included in the rad-score

**
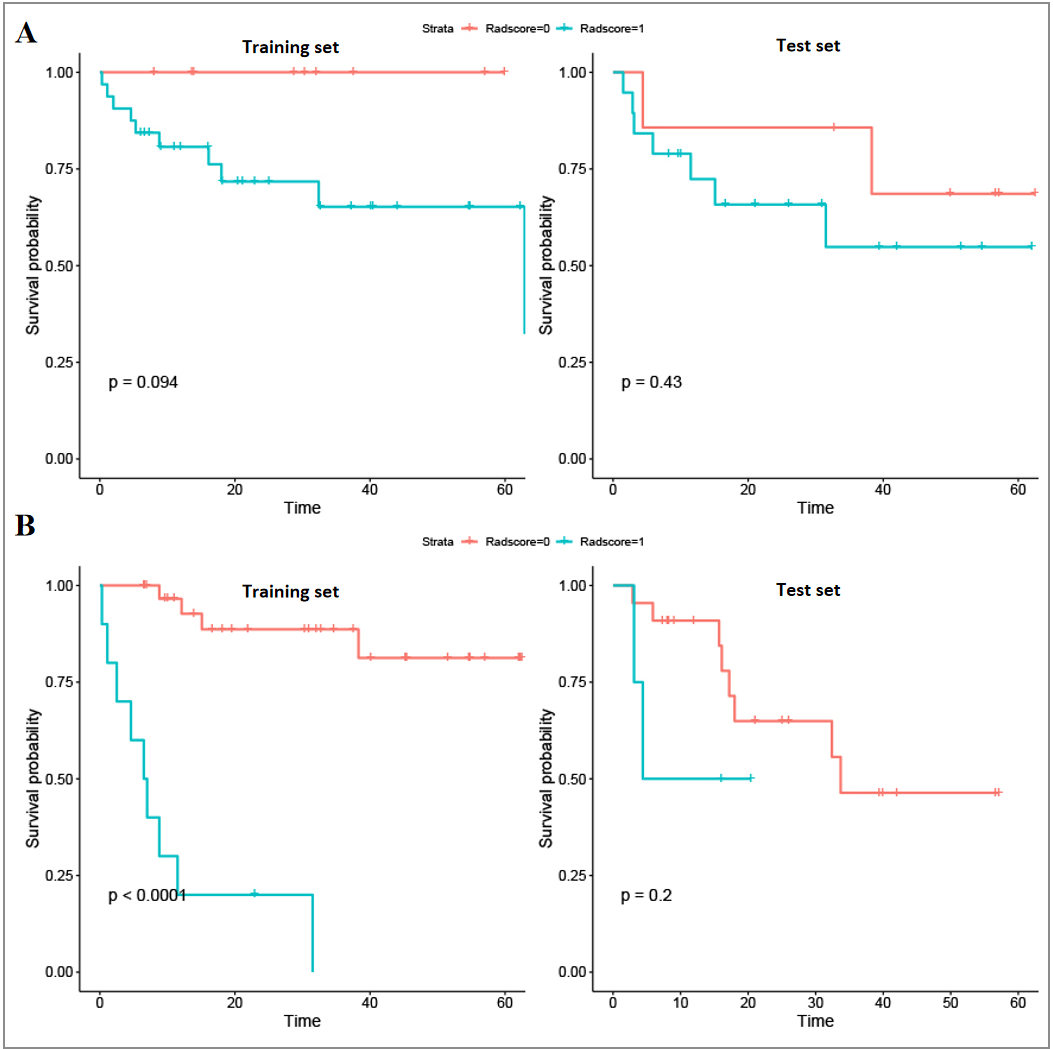
**

**Figure S2.** Kaplan–Meier survival curves of patients grouped according to rad-score in the training and test sets. Kaplan-Meier survival analysis was performed in the training set and test set based on rad-score derived from positron emission tomography (A) and computed tomography (B). **(A)** Patients grouped according to rad-score derived from positron emission tomography showed no significant association with progression-free survival in training set and test set. **(B)** A significant association of the rad-score derived from computed tomography with progression-free survival was shown in the training set but not the test set


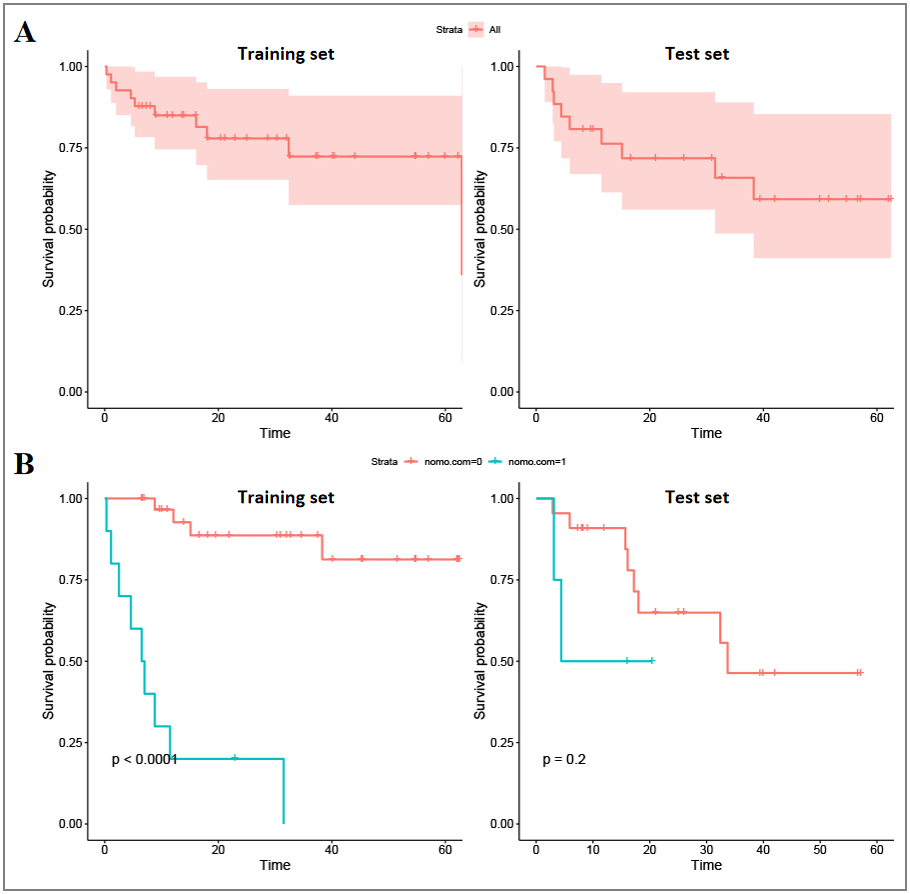


**Figure S3.** Kaplan-Meier survival analysis of the integrated clinical radiomics (ICR) model in training and test sets. Kaplan-Meier survival analysis was performed in the training and test sets based on ICR model from positron emission tomography (A) and computed tomography (B). **(A)** Failed to screen out suitable features combination from rad-score derived from positron emission tomography and clinical features to construct ICR model, and further analyses were unable to be performed. **(B)** A significant association of the ICR model with progression-free survival was shown in the training set, but not the test set
